# Trp207 regulation of voltage-dependent activation of human H_v_1 proton channel

**DOI:** 10.1016/j.jbc.2024.105674

**Published:** 2024-01-23

**Authors:** Lu Zhang, Xin Wu, Xinyu Cao, Khushi Rao, Liang Hong

**Affiliations:** 1 Department of Medicine, University of Illinois at Chicago, Chicago, Illinois, USA; 2 Department of Biomedical Engineering, University of Illinois at Chicago, Chicago, Illinois, USA; 3 Department of Biological Sciences, Purdue University, West Lafayette, Indiana, USA; 4 Department of Physiology and Biophysics, University of Illinois at Chicago, Chicago, Illinois, USA

**Keywords:** H_v_1, voltage-gated ion channel, proton channel, voltage-sensing domain, channel activation

## Abstract

In voltage-gated Na^+^ and K^+^ channels, the hydrophobicity of noncharged residues in the S4 helix has been shown to regulate the S4 movement underlying the process of voltage-sensing domain (VSD) activation. In voltage-gated proton channel H_v_1, there is a bulky noncharged tryptophan residue located at the S4 transmembrane segment. This tryptophan remains entirely conserved across all H_v_1 members but is not seen in other voltage-gated ion channels, indicating that the tryptophan contributes different roles in VSD activation. The conserved tryptophan of human voltage-gated proton channel H_v_1 is Trp207 (W207). Here, we showed that W207 modifies human H_v_1 voltage-dependent activation, and small residues replacement at position 207 strongly perturbs H_v_1 channel opening and closing, and the size of the side chain instead of the hydrophobic group of W207 regulates the transition between closed and open states of the channel. We conclude that the large side chain of tryptophan controls the energy barrier during the H_v_1 VSD transition.

The voltage-gated ion channels, including Na_v_, K_v_, Ca_v_, and H_v_1 proton channels, all contain voltage-sensing domains (VSDs) that are responsible for detecting changes in membrane potential in the cells (1, 2, 3). The VSDs of voltage-gated ion channels are made of four transmembrane segments (S1 through S4). The S4 helix contains several positively charged residues located at every third position. To respond to the changes in membrane potential, the S4 helix undergoes transmembrane movement that is mediated by these charged residues (4, 5, 6). Moreover, other noncharged residues in the S4 helix have been shown to modify the S4 movement, and the hydrophobicity of noncharged residues regulates the process of voltage-dependent activation of the channels (7, 8, 9). In the Shaker K_v_ channel, replacements of hydrophilic residues at position 361 (L361K and L361R) in the S4 helix profoundly shifted the *G-V* curve toward negative voltages, and hydrophilic replacements at this position produced faster activation kinetics (8). In the Na_v_1.4 channel, substitution of hydrophobic residues in the S4 helix of domains I, II, and III has been shown to regulate the voltage sensor movement and alter steady-state activation and inactivation curves (7).

In the voltage-gated proton channel family, there is a bulky noncharged residue tryptophan located at the S4 helix, which remains highly conserved across all H_v_1 channels (Fig. 1*A*) (10, 11). Despite progress in the functional characterization of noncharged residues in the S4 helix of Na_v_ and K_v_ channels (7, 8, 9), the role of S4 noncharged residues in H_v_1 channel voltage-dependent activation remains unclear. In addition, the bulky tryptophan is highly conserved in the H_v_1 family (Fig. 1*A*) but is not seen in other voltage-gated ion channels (Fig. S1), indicating that it might contribute different roles in VSD activation.

**Figure 1:**
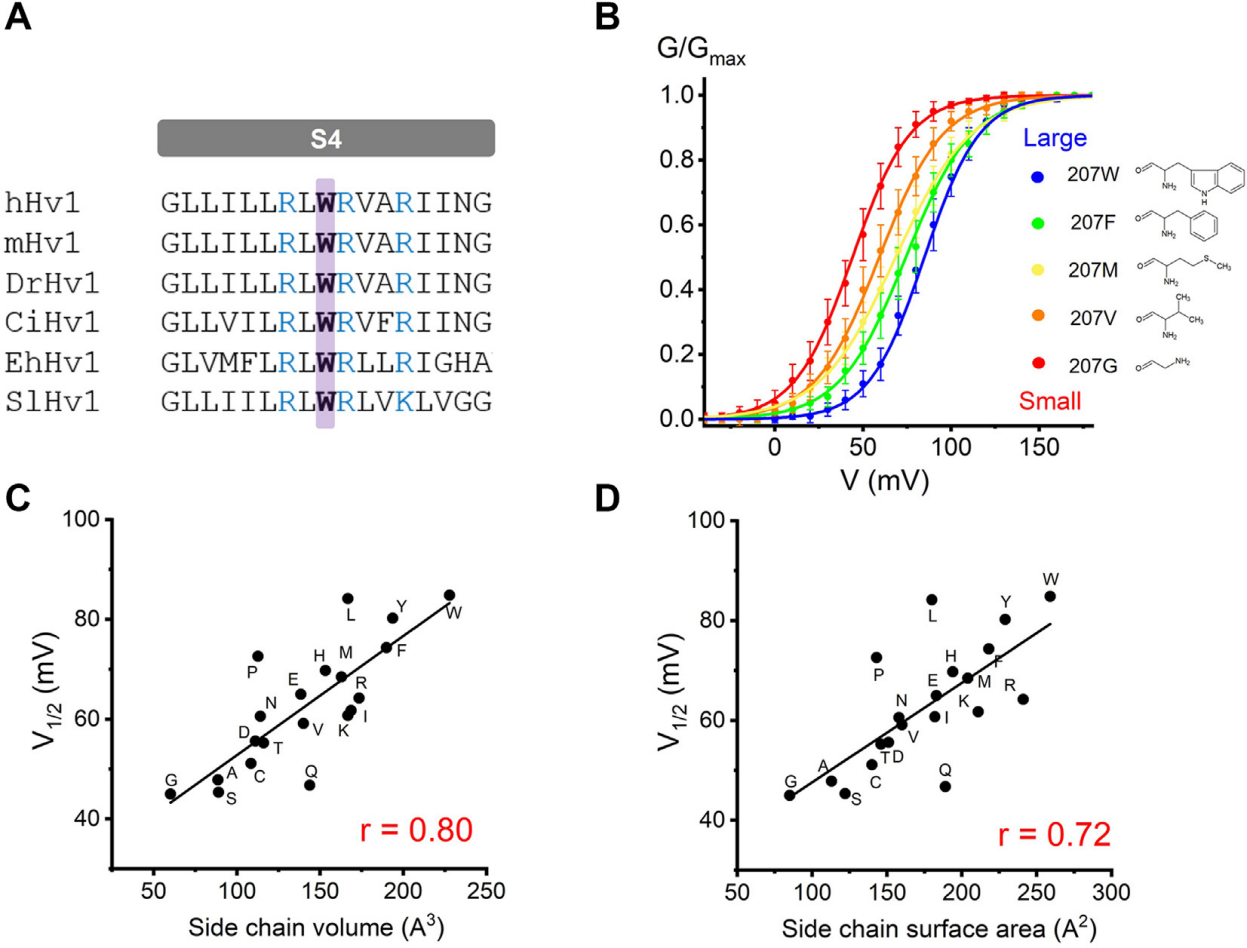
**The bulky side chain at position 207 regulates H**_**v**_**1 voltage-dependent activation.***A*, sequence alignment of the S4 transmembrane segment of H_v_1 proton channels from human (hH_v_1), mouse (mH_v_1), zebrafish (DrH_v_1), *Ciona intestinalis* (CiH_v_1), Emiliana (EhH_v_1), and *Suillus luteus* (SlH_v_1). Highly conserved tryptophan (W) is highlighted in *purple* and *bold font*. W corresponds to Trp207 in hH_v_1. The positive S4 residues are highlighted in *blue*. *B*, *G-V* curves for the monomer hH_v_1 proton channel W207 mutations colored from *blue* to *red* when the side chain of the substituted side chain decreases, and structures of substituted residues are shown in inset. Only several W207 mutations are shown for clarity, and Table S1 summarized all W207 mutations, n = 4 to 8 for each mutation. *C* and *D*, the *V*_*1/2*_ values obtained from *G-V* curves are plotted with size of the substituted side chain at position W207, using either side chain volume (*C*) or side chain surface area (*D*). *Black lines* indicate fits of the data to a linear function in (*C* and *D*), and r values are presented in *red* in each panel.

The highly conserved tryptophan of the human H_v_1 channel (hH_v_1) is W207. In Na_v_ and K_v_ channels, the hydrophobic group of noncharged residues in S4 helix significantly influences the VSD activation (7, 8, 9). To assess whether the hydrophobic group of W207 regulates hH_v_1 VSD activation as well, we introduced mutations in the W207 by substituting the other 19 amino acids. In contrast to findings in other channels, we showed that the size of the side chain instead of the hydrophobic group at position W207 is essential for the H_v_1 proton channel voltage-dependent activation. We further showed that small residues replacement at position 207 exhibited faster activation and deactivation kinetics, suggesting that the presence of the natively bulky tryptophan slows the gating process and controls the transition between closed and open states of the channel. We proposed a simple-state model of H_v_1 VSD transition and discussed the conserved Trp regulation of H_v_1 VSD activation.

## Results

### Effects of W207 mutations on the voltage-dependent activation of human H_v_1 channel

The H_v_1 channel has been shown to form dimers in which two VSD subunits are held together by a C-terminal coiled-coil domain (12, 13, 14), and deletion of the coiled-coil domain results in a monomeric form of the channel (15, 16, 17). H_v_1 VSD subunits were reported to be allosterically coupled and work cooperatively (18, 19). To eliminate the influence of cooperation between subunits, we generated monomeric hH_v_1 by deletion of coiled-coil domain.

**Figure 2:**
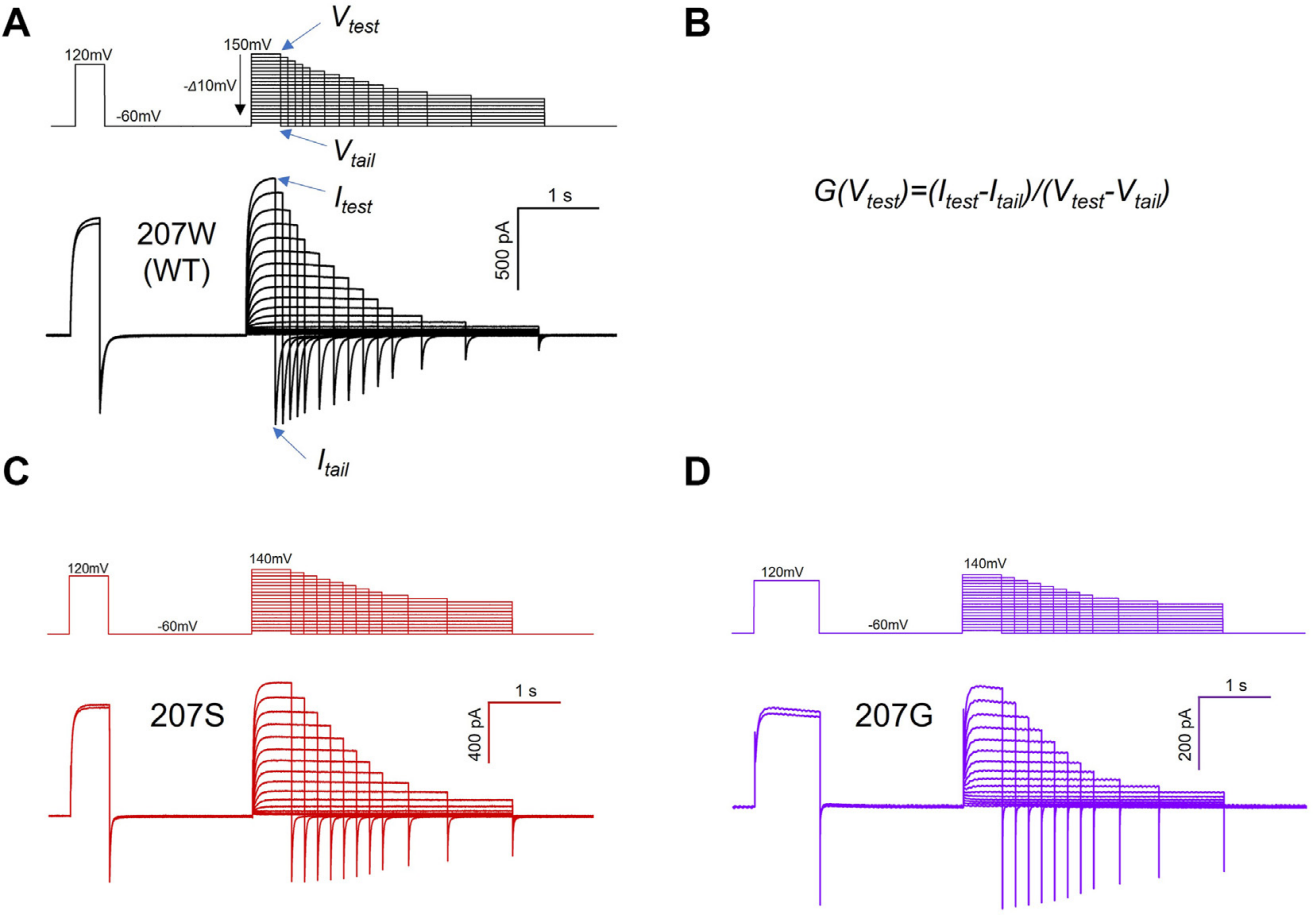
**G-V measurements from monomer H**_**v**_**1 channel currents.***A*, representative currents were recorded in HEK293 cells expressing monomer WT (207W) H_v_1 channel, pH_i_ = pH_o_ = 6.0. The corresponding pulse protocols are shown above the current traces. Currents were measured from a holding potential of −60 mV to a first prestep (+120 mV) and a second test-step (ranging between +150 and −50 mV in 10 mV steps) with rest intervals at −60 mV. Currents after depolarization in the prestep to +120 mV were used to correct for current rundown. For clarity, only the first and last traces elicited by the depolarization prestep are shown. The *blue arrows* indicate the parameters used for the channel conductance analysis. *B*, the channel conductance is determined by the equation: *G(V*_*test*_*) = (I*_*test*_*-I*_*tail*_*)/(V*_*test*_*-V*_*tail*_*)*, where *I*_*test*_ is the current at *V*_*test*_, measured at the end of the depolarization step, and *I*_*tail*_ is the current at *V*_*tail*_, measured at the beginning of the repolarization step. *C* and *D*, representative currents were recorded in HEK293 cells expressing monomer H_v_1 mutations 207S (*C*) and 207G (*D*), pH_i_ = pH_o_ = 6.0. The corresponding pulse protocols are shown above the current traces.

We introduced mutations at position 207 in monomeric H_v_1 to investigate effects of the conserved tryptophan on the H_v_1 voltage-dependent activation. The representative currents recorded in HEK293 cells expressing monomer human H_v_1 (hH_v_1) 207W (wildtype) and 207 mutation channels are shown in Figure 2. In the recording, proton currents were measured from a holding potential of −60 mV to a first prestep and a second test-step with rest intervals at −60 mV. The proton channel conductance is determined by the equation: *G(V*_*test*_*) = (I*_*test*_*-I*_*tail*_*)/(V*_*test*_*-V*_*tail*_*)*, where *I*_*test*_ is the current at *V*_*test*_, measured at the end of the depolarization step, and *I*_*tail*_ is the current at *V*_*tail*_, measured at the beginning of the repolarization step. Currents after depolarization in the prestep were used to correct for current rundown.

It was shown that all W207 mutations produced left-shifted *G-V* relationships (Fig. 1*B*, Table S1), suggesting that mutations at position 207 perturb the channel voltage-dependent gating and that the conserved tryptophan is essential for H_v_1 VSD activation. To investigate the relationships between the H_v_1 voltage dependence of activation and amino acid mutations' physicochemical properties, we conducted a linear correlation analysis. We determined the midpoint voltage (V_1/2_) from the fitted *G-V* curve to measure the voltage dependence of activation parameter. The physicochemical properties we considered for amino acids were side chain size and hydrophobicity (20, 21, 22).

**Figure 3:**
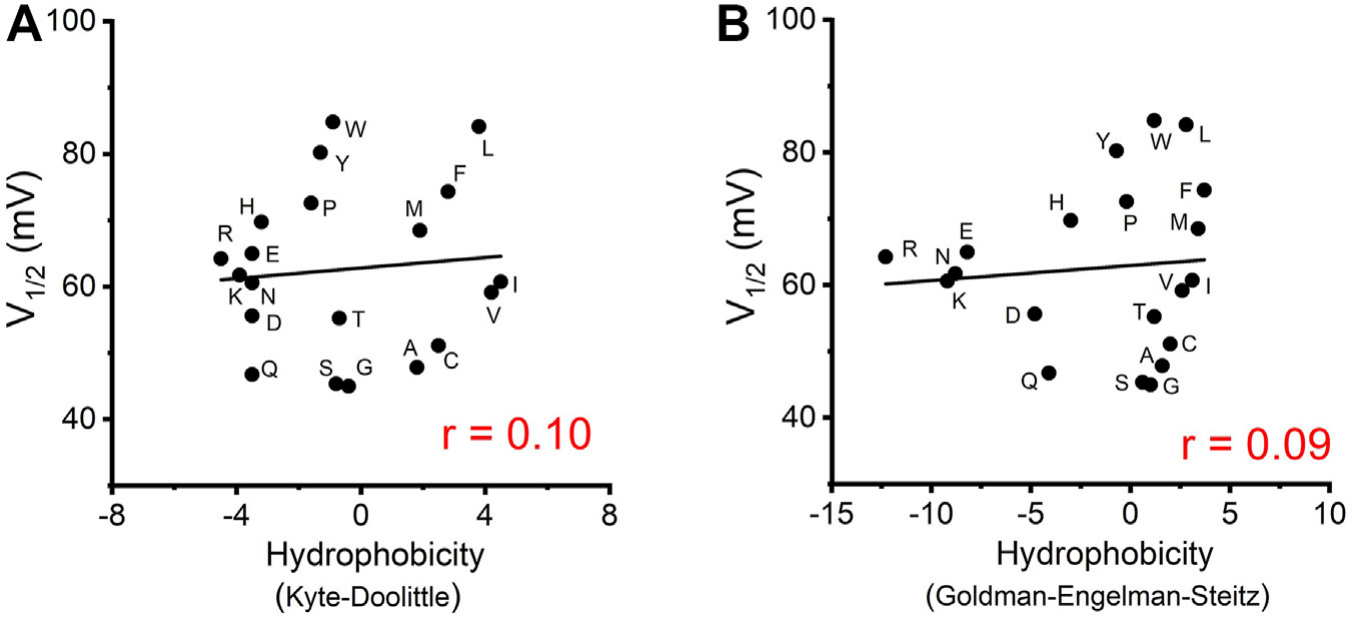
**The hydrophobic group at position 207 does not correlate with H**_**v**_**1 voltage-dependent activation.***A* and *B*, the *V*_*1/2*_ values obtained from *G-V* curves are plotted as a function of hydrophobicity of the substituted side chain at position W207, using either *Kyte-Doolittle* hydrophobicity scale (*A*) or *Goldman-Engelman-Steitz* hydrophobicity scale (*B*). *Black lines* indicate fits of the data to a linear function, r values are presented in *red* in each panel.

Our analysis revealed that the hydrophobicity of the side chain had no significant correlation with V_1/2_ (Fig. 3). However, we observed a robust linear relationship between the voltage dependence of H_v_1 VSD and the side chain surface area or volume (Fig. 1, *C* and *D*). These results indicated that the size of the side chain at position 207 was closely associated with V_1/2_, with a bulky residue at position W207 being critical for channel function.

W207 mutations have been shown to perturb *Δ*pH-dependent gating at lower pH_o_ (23). Our results showed that, in monomer WT H_v_1 channel, the *G-V* shifts between pH_o_ = 7/pH_i_ = 6 and pH_o_ = 6/pH_i_ = 6 is 38 ± 5 mV, whereas the shift is 21 ± 6 mV in W207A and 19 ± 5 mV in W207G (Fig. S3). The results are consistent with previous findings that W207 mutants compromise the *Δ*pH-dependent gating of the H_v_1 channels (23).

### Effects of small side chain substitutions of W207 on the open and closed states of H_v_1

**Figure 4:**
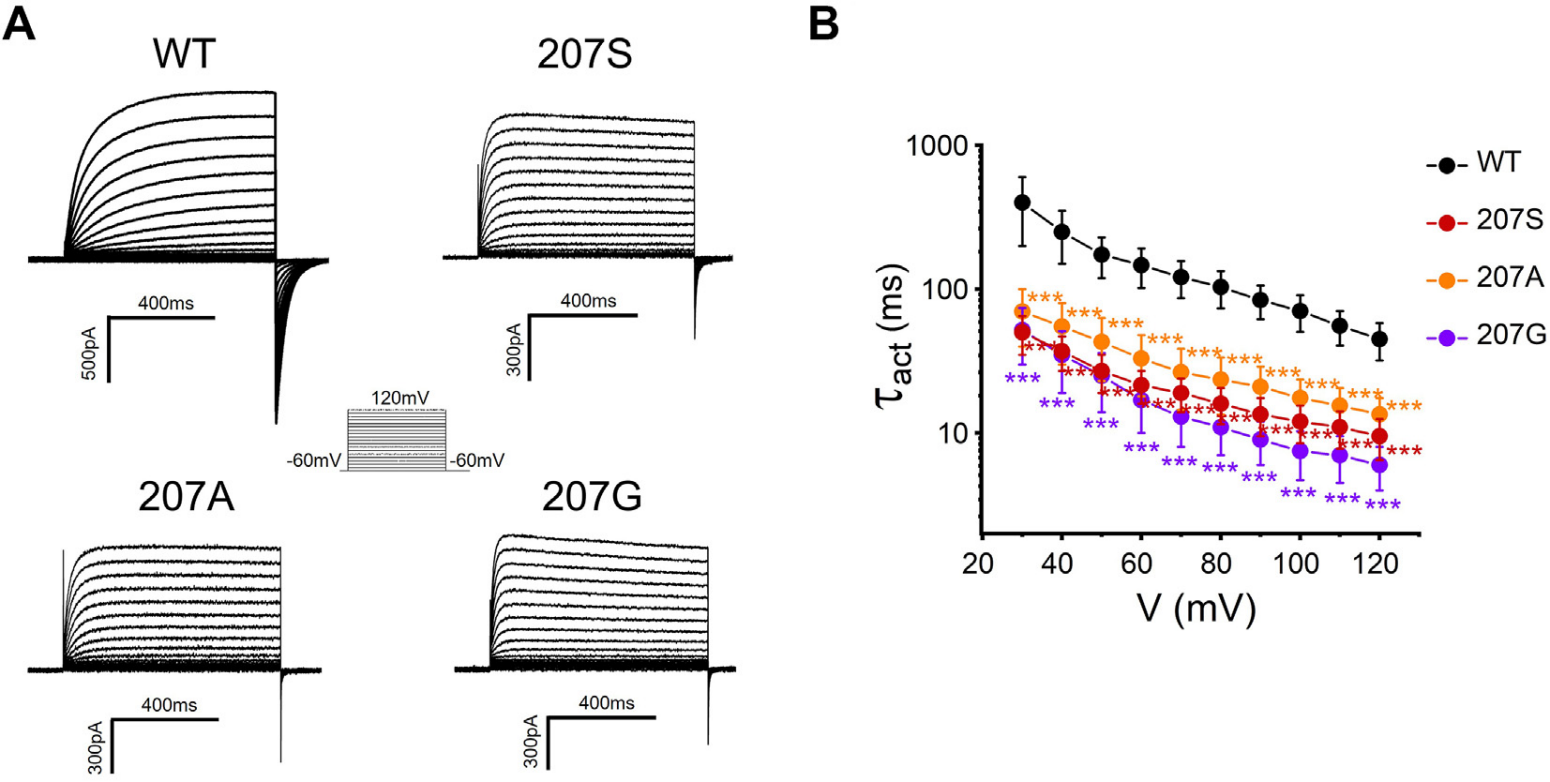
**Effects of small side chain substitutions of W207 on the open state of the monomer H**_**v**_**1 channel.***A*, representative rising currents recorded from WT (207W), 207S, 207A, and 207G. Currents were measured from a holding potential of −60 mV to test potentials ranging between −60 and +120 mV in 10 mV steps. *B*, the channel opening time constant *Ʈ*_*act*_ in WT or W207 mutations. *Ʈ*_*act*_ was obtained from exponential fit to rising currents, n = 3 to 6 for each group. *Ʈ*_*act*_ between W207 mutation and WT were compared statistically using two-tailed test (∗∗∗*p* < 0.001).

To investigate how the large side chain of W207 affects the H_v_1 voltage-dependent activation, we substituted the tryptophan with a small residue alanine at position 207 (207A). Mutation 207A exhibited significantly faster activation kinetics compared with wildtype channel (207W), suggesting that 207A mutation favors the open state of the channel (Fig. 4*A*). We then introduced two more small residues at position 207 (207G and 207S), and both were shown to facilitate channel opening with faster activation kinetics (Fig. 4*B*). These results indicated that small residue replacements at position 207 are likely to reduce an energy barrier for the channel activation and make the channel easier to open, accounting for faster activations in the mutated channels (207A, 207G, and 207S).

**Figure 5:**
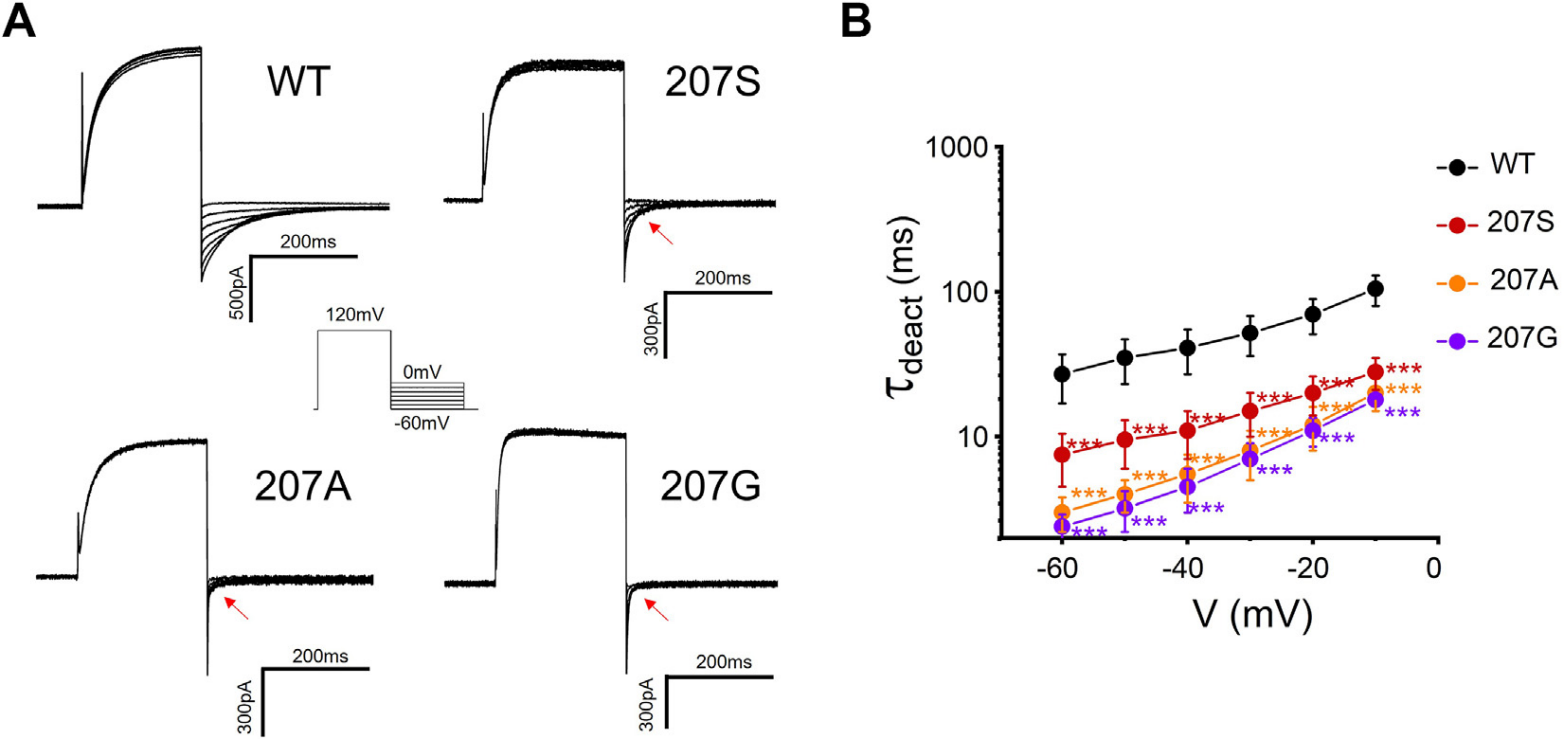
**Effects of small side chain substitutions of W207 on the closed state of the channel.***A*, representative tail currents recorded from WT (207W), 207S, 207A, and 207G. The tail currents were elicited by a prepulse to 120 mV, in 10 mV decrements from 0 to −60 mV. *Red arrows* indicate faster deactivation of W207 mutations. *B*, the deactivation (channel closing) time constant *Ʈ*_*deact*_ in WT or W207 mutations. *Ʈ*_*deact*_ was obtained from exponential fit to the tail currents, n = 3 to 6 for each group. *Ʈ*_*deact*_ between W207 mutation and WT were compared statistically using two-tailed test (∗∗∗*p* < 0.001).

The effects of a small side chain at position 207 on the closed state of the channel were further investigated. The 207A mutation showed faster deactivation kinetics (Fig. 5*A*). The channel closing time constant Ʈ_deact_ values were determined, and 207A took less time to close compared with WT (Fig. 5*B*). Similar results have been observed in the other two small residues replacement at position 207 (207G, 207S), and both 207G and 207S altered the closing time constant Ʈ_deact_ decreasing the values at given membrane potentials (Fig. 5*B*). This indicates that small residue replacements at position 207 also reduce an energy barrier for channel deactivation and facilitate channel closing.

**Figure 6:**
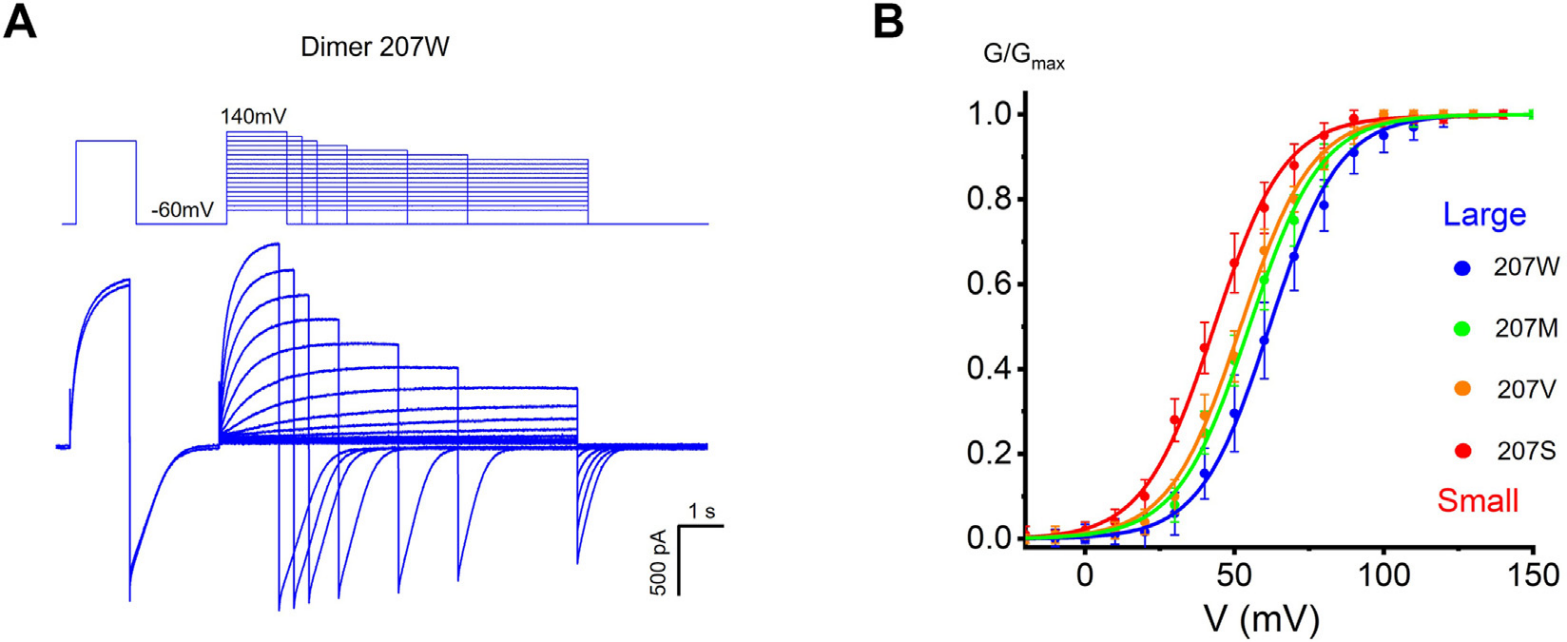
**Effects of side chain at position 207 on dimer H**_**v**_**1 channel voltage-dependent activation.***A*, representative currents were recorded in HEK293 cells expressing dimer WT H_v_1 (207W) channel, pH_i_ = pH_o_ = 6.0. For clarity, only the first and last traces elicited by the depolarization prestep are shown. The corresponding pulse protocols are shown above the current traces. *B*, *G-V* curves for the dimer H_v_1 proton channel W207 mutations colored from *blue* to *red* when the side chain of the substituted side chain decreases, n = 4 to 7 for each group.

**Figure 7:**
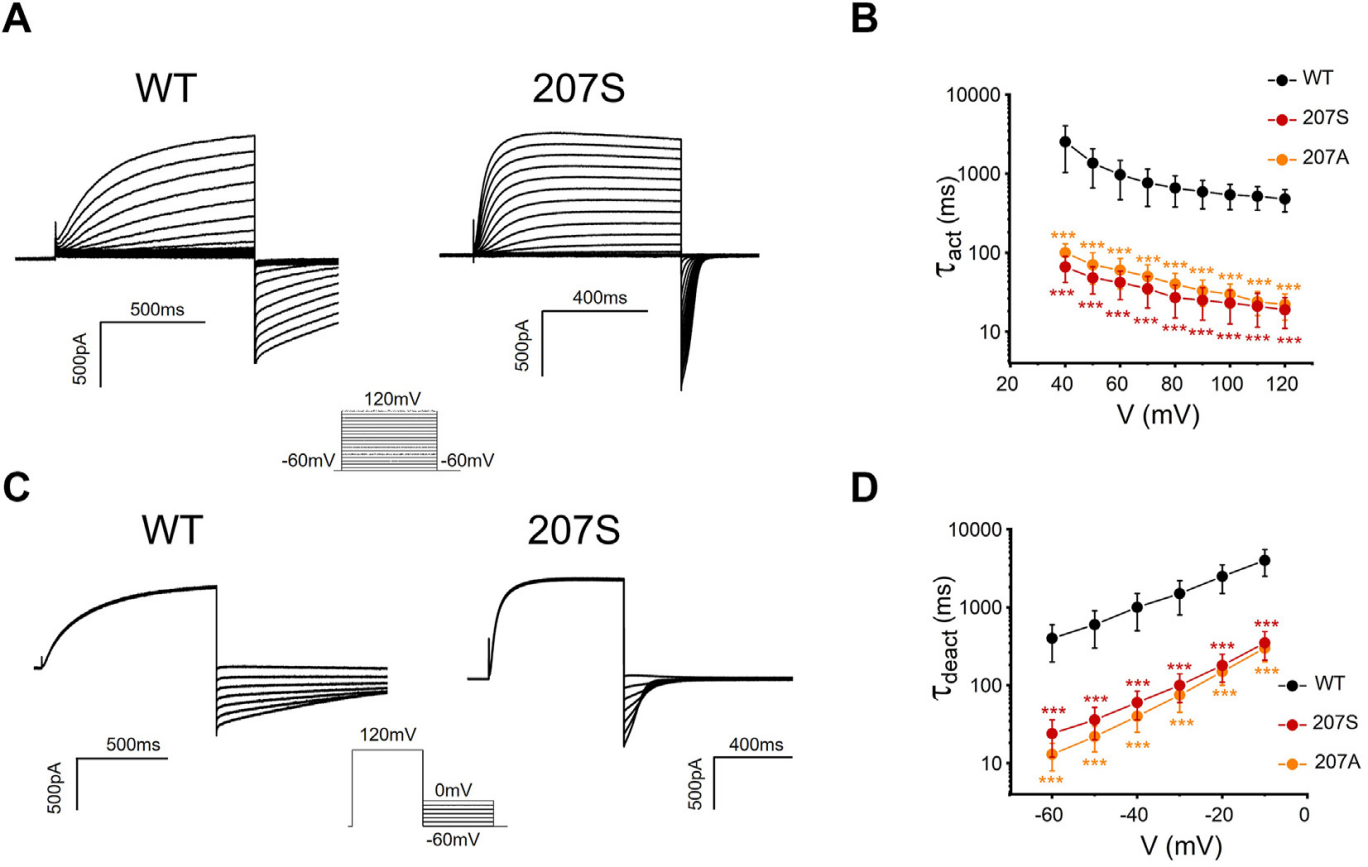
**Effects of small side chain substitutions of W207 on the open and closed state of the dimer H**_**v**_**1 channel.***A*, representative rising currents recorded from dimer WT and 207S channels. Currents were measured from a holding potential of −60 mV to test potentials ranging between −60 and +120 mV in 10 mV steps. *B*, the channel opening time constant *Ʈ*_*act*_ in dimer H_v_1 channels. *Ʈ*_*act*_ was obtained from exponential fit to rising currents. *Ʈ*_*act*_ between W207 mutation and WT were compared statistically using two-tailed test (∗∗∗*p* < 0.001). *C*, representative tail currents recorded from dimer WT and 207S channels. The tail currents were elicited by a prepulse to 120 mV, in 10 mV decrements from 0 to −60 mV. *D*, the deactivation (channel closing) time constant *Ʈ*_*deact*_ in dimer H_v_1 channels. *Ʈ*_*deact*_ was obtained from exponential fit to the tail currents, n = 4 to 6 for each group. *Ʈ*_*deact*_ between W207 mutation and WT were compared statistically using two-tailed test (∗∗∗*p* < 0.001).

We further evaluated dimer hH_v_1 channels. It was shown that the bulky side chain at position 207 regulates dimer hH_v_1 voltage-dependent activation as well, and smaller side chain substitutions of Trp consistently produce left-shifted *G-V* relationships (Fig. 6). Moreover, dimer channel W207 mutations exhibited much faster activation and deactivation kinetics compared with dimer wildtype channel (Fig. 7), suggesting that small side chain substitutions of Trp favor the open and closed state of the dimer channel. In other words, small residue replacement reduces an energy barrier during the H_v_1 channel voltage-dependent activation.

Taken together, these results suggest that the endogenous residue tryptophan at position 207 increases the energy barrier that makes the channel neither easily open nor easily closed.

### State model of W207 mutation regulation of H_v_1 VSD transitions

**Figure 8:**
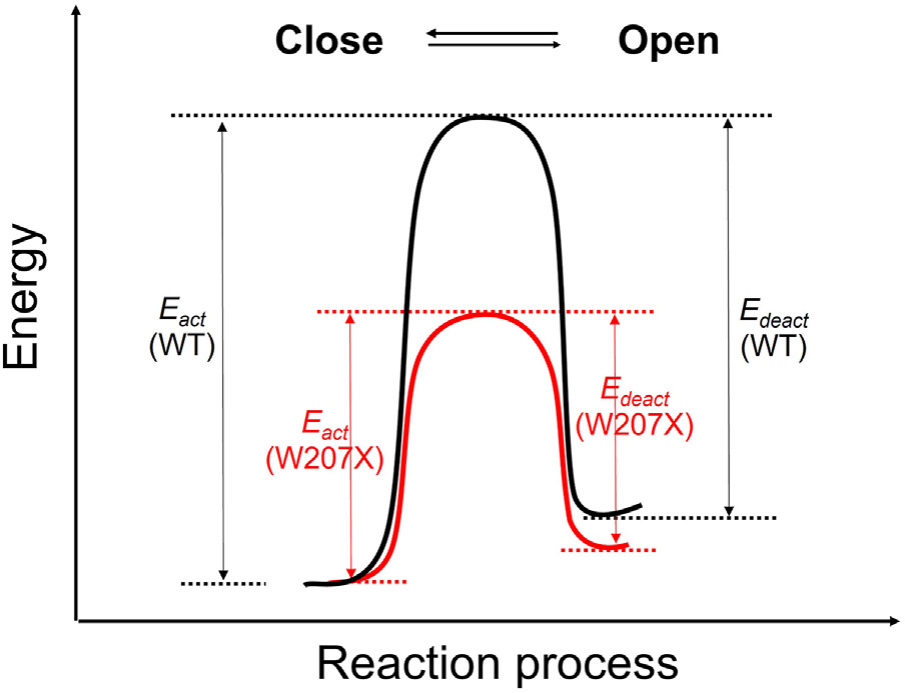
**State model of W207 mutation regulation of H**_**v**_**1 VSD transitions.** The *black lines* represent the voltage-dependent activation for bulky side chain Trp (W) at position 207 (*i.e*., wildtype H_v_1 channel), and *red lines* are changes mediated by the small side chain (X) substitutions at position 207 (W207X). The reduced energy barrier caused by W207X is satisfied by decreased activation kinetic *E*_*act*_(W207X) and decreased deactivation kinetic *E*_*deact*_(W207X).

To describe the effects of small residue replacements at position 207 on the H_v_1 VSD voltage-dependent activation, we proposed a simple-state model of H_v_1 VSD transitions. In the model, the small residue replacements (W207X) decreased the energy barrier underlying the transition between closed and open states of the channel (Fig. 8).

The effects of W207X mutations on the closed state were satisfied by decreasing the relative depth of the energy well for the “Close” state and decreasing the energy barrier for the Close - > Open transition to allow for the faster deactivation (Figs. 5*A* and 8). W207X mutations’ influences on the open state could also be satisfied by decreasing the relative depth of the energy well for the “Open” state, which is achieved by the reduced energy barrier for the Open - > Close transition to interpret the faster activation kinetics (Figs. 4*A* and 8). A smaller *ΔG*_*0*_ (which is a free energy difference between the closed and open states of the activation, Table S1) for W207X was consistent with decreased activation *E*_*act*_(W207X) (Fig. 4*B*) and reduced deactivation kinetics *E*_*deact*_(W207X) (Fig. 5*B*).

## Discussion

In the H_v_1 family, there is a bulky residue tryptophan located at the S4 segment, which remains entirely conserved across all H_v_1 members. This highly conserved Trp has been shown to play important roles in regulating dimer coupling and pH gradient-dependent gating (18, 23, 24). Here, we showed that the Trp strongly modifies the process of H_v_1 voltage-dependent activation. The large side chain of the Trp controls the transition between closed and open states of the H_v_1 channel and regulates the energy barrier during the H_v_1 VSD transition.

To determine the effects of Trp on H_v_1 VSD voltage-dependent activation, we introduced other 19 residues at position 207 in monomer hH_v_1 channel. We determined the *G-V* curves of W207 mutations and analyzed the effects of physicochemical properties of W207 mutations on the voltage-dependent activation. In contrast to the findings that the hydrophobicity of noncharged residues in the S4 helix of Na_v_ and K_v_ channels play roles in regulation of VSD activation (7, 8), there were no significant correlations between the hydrophobicity of side chain and the V_1/2_ in H_v_1. However, replacement of smaller residues at position 207 consistently shifts the *G-V* curve toward more negative voltages, indicating that large size of side chain at position 207 is crucial for the H_v_1 channel voltage-dependent activation. Additionally, we notice that some amino acids containing rigid rings (*e.g.*, 207P, 207W, 207F, and 207Y) generate relatively higher V_1/2_ (Table S1). Although the leucine (L), which does not have rigid rings, also generates a greater V_1/2_, we cannot exclude the possible contributions of rigid rings to the channel activation, which might increase the energy cost.

We replaced the tryptophan (W) with the three smallest residues (A, S, G) to determine how the largest residue (W) at position 207 affects hH_v_1 VSD activation, and all small residues exhibited faster activation and deactivation kinetics, indicating that small residues replacement facilitate channel opening and closing. These results suggest that the endogenous residue tryptophan increases the energy barrier that makes the channel neither easily open nor easily closed. We observed that the whole cell currents carried by W207 mutations are less than wildtype monomer channel (Fig. 4). We considered an explanation that W207-mutated channel might produce either relatively smaller single channel conductance and/or lower expression of the cell membrane that overcomes the advantage of open probability to influence the whole cell current amplitude.

H_v_1 is mainly expressed in nonexcitable cells such as epithelial cells, sperm cells, and white blood cells, in which H_v_1 is implicated in pH regulation in airway epithelium and sperm cells and reactive oxygen species production in phagocytes (2, 3). The pH is determined by the H^+^ concentration, and an alteration of H^+^ concentration causes abnormal local pH that influences cell physiology. Overactivity of the H_v_1 channel can result in low concentration of intracellular H^+^ and lead to cellular acid–base imbalances (2). The presence of large tryptophan in the H_v_1 channel can prevent frequent transitions between closed and open states of the channel and protect against the overactivity of H_v_1, and this function might manipulate H_v_1 activity to coordinate the regulation of pH homeostasis in white blood cells, sperm cells, epithelial cells, and other cells where H_v_1 expressed and maintain normal physiological functions in these cells.

The H_v_1 channel function is regulated by pH gradient (*Δ*pH). It was reported that the *G-V* of H_v_1 channel shifts around 40 mV/unit change in *Δ*pH, regardless of whether pH_o_ or pH_i_ is changed (25). W207 mutations have been shown to influence *Δ*pH-dependent gating at lower pH_o_, supporting the existence of distinct internal and external pH sensors (23). In the present study, W207 mutations exhibited less *G-V* shifts compared with wildtype channel in the same pH gradient, which is consistent with previous findings that W207 mutations compromise the *Δ*pH-dependent gating of the H_v_1 channels (23). W207 mutations might generate allosterically conformational effects on the pH sensors to perturb the *Δ*pH-dependent gating of the channel.

The H_v_1 channel has been shown to form dimers in which two VSD subunits are held together by a C-terminal coiled-coil domain (12, 13, 14). Studies have found that the two H_v_1 subunits gate cooperatively, and it was shown that the opening of one subunit substantially increases the probability of the other subunit to open (19). Okuda *et al.* discovered a molecular mechanism by which two H_v_1 subunits cooperate with each other (18). It was shown that the two H_v_1 S4 helices within the dimer directly cooperate *via* a π-stacking interaction between Trp residues at the middle of each segment. To delineate the interaction between two H_v_1 subunits, Okuda *et al.* performed a scanning mutagenesis in the S4 transmembrane helices of the H_v_1 channel and showed that the aromatic-aromatic interaction mediated by two Trps plays a role in regulation of the dimer’s cooperativity. Okuda *et al.* showed that replacement of Trp in some positions of the S4 helix (*e.g.*, 250W, 253W, 257W in Ciona Hv1) produced stronger effects for deactivation. The analysis of interaction energies for the positions 250, 253, and 257 in the S4 helix showed strong interaction of the two Trp residues in two subunits during the deactivation phase, suggesting that positions 250, 253, and 257 play crucial roles in regulating two Hv1 subunits interaction during gating cooperativity (18).

In the present study, we focus on the role of Trp in regulating H_v_1 voltage sensor transition in each VSD subunit. Our results showed that Trp modifies the process of H_v_1 voltage-dependent activation (*i.e.*, a process by which the channel transitions between its closed and open states). We performed mutagenesis at position Trp207 in human H_v_1 channel and found that replacement of small residues at position 207 exhibited faster gating kinetics, in other words, the endogenous Trp at the position 207 presented much slower deactivation and activation compared to the ones of small residues substitution, indicating that the Trp at position 207 plays a role in the regulation of Hv1 channel gating. To date, although several models of voltage sensing, including transporter model, helical screw model, and paddle model, were proposed to describe the movement of VSD S4 helix in voltage-gated Na^+^, Ca^+^, and K^+^ channels (26), so far, the way of the movement of S4 helix in H_v_1 VSD subunit is not yet clear. Our results indicated that the bulky side chain of Trp mediates the movement of H_v_1 VSD S4 helix that might undergo rotating and translate along its axis to move across the transmembrane electric field, similar with helical screw model in other ion channels. The small side chain at position 207 reduces the barrier during the process of H_v_1 S4 helix rotation and translation in H_v_1 VSD, accounting for fast activation and deactivation of the channel when W207 mutated to small residues.

**Figure 9:**
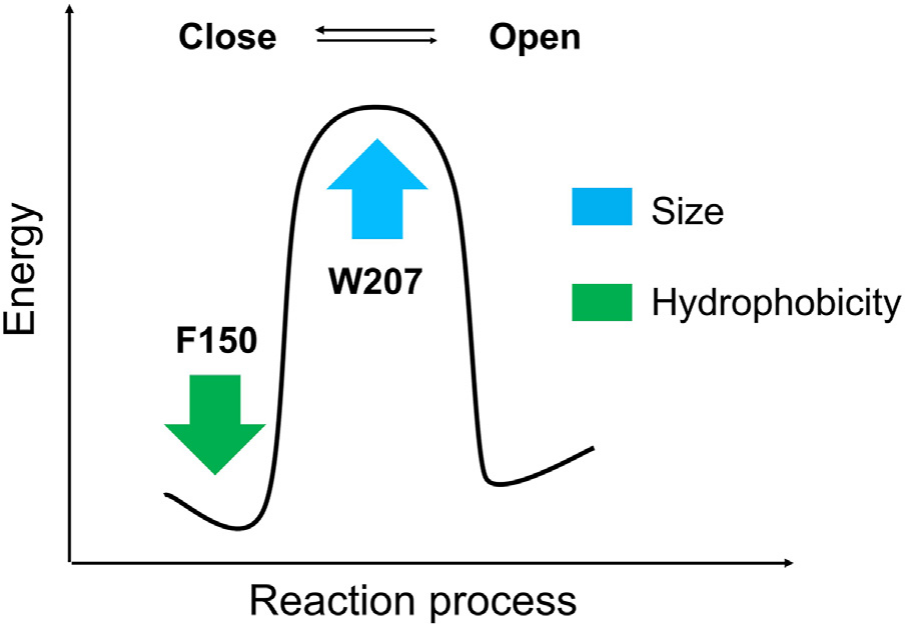
**Contributions of conserved residues to the H**_**v**_**1 VSD energy landscape.** Previous study showed that increasing the hydrophobicity of residue at position F150 (*green arrow*) stabilizes the resting state relative to active state. Present study indicates that increasing the size of the side chain at position W207 (*blue arrow*) is essential to control H_v_1 VSD energy barrier. VSD, voltage-sensing domain.

We previously found that a highly conserved residue F150 is important for the hH_v_1 channel function and that the hydrophobic group of F150 stabilizes the resting H_v_1 VSD (27). Here, we showed that another highly conserved residue W207 regulates the energy barrier during the H_v_1 VSD transition. In contrast to the contributions of hydrophobicity of F150 to the channel function, the large size of side chain of W207 is essential for the H_v_1 VSD activation, in which it controls the transition between closed and open states of the channel (Fig. 9). The H_v_1 proton channel is known to play a role in reactive oxygen species generation and the regulation of pH homeostasis (25, 28, 29, 30, 31, 32). Exploring how the conserved residues including W207 regulates the process of H_v_1 voltage-dependent activation will provide insights into the development of targeted reagents modulating H_v_1 activity (33).

## Experimental procedures

### Mutagenesis

Recombinant human H_v_1 channels were subcloned in the pNICE vector. Monomeric hH_v_1 was achieved by introducing a stop codon at position 224 (S224_stop_), which deletes coiled-coil domains essential for H_v_1 dimer formation (15). Single-point mutations were introduced with standard PCR techniques (34, 35). PCR primers were purchased from IDT DNA Technologies. Custom-designed primers for mutagenesis of hH_v_1 constructs were included in Table S2. H_v_1 mutation was introduced to the template plasmid using primers in a PCR protocol. The PCR cycles were initiated at 98 °C for 1.5 min, followed by 25 amplification cycles. Each amplification cycle consisted of 98 °C (20 s), 60 °C (30 s), and 72 °C (5 min). The cycles were finished with an extension step at 72 °C for 15 min, followed by 4 °C for 30 min. After template plasmid was removed by DpnI (NEB, R0176S), the Stbl2 competent cells (Invitrogen,10268019) were transformed with the PCR product. Plasmids were isolated from Stbl2 cells with resulting colonies using QIAprep Spin Miniprep Kit (Qiagen, 27106). Mutation confirmed by the DNA sequencing was used for subsequent transfection.

### Cell culture and H_v_1 transfection

HEK293 cells (Sigma-Aldrich) were reseeded on coverslips containing Dulbecco modified Eagle’s medium supplemented with 10% FBS, 100 U/ml penicillin, and 100 μg/ml streptomycin at 37 °C under 5% CO_2_. Cells were transiently transfected with human H_v_1 and GFP cDNA plasmids after growth to ∼70% confluence. The HEK-293 cells were transiently transfected with 2 μg of the cDNA encoding H_v_1 construct and 0.25 μg of a plasmid encoding GFP using Lipofectamine 3000 reagent (Invitrogen) according to the manufacturer’s protocol. The mixture was then added to the culture dish, and the cells were incubated at 37 °C for 24 h before the electrophysiology studies were conducted. A coverslip with HEK293 cells was placed in a recording chamber containing bath solution on the stage of a fluorescence microscope (Olympus), and the transfected cells detected by the fluorescent signal emitted from GFP were applied for electrophysiological measurements. Patch clamp experiments were conducted 1∼3 days after transfection.

### Electrophysiological measurements and analysis

H_v_1 proton currents were recorded with whole-cell configuration using an Axopatch 200B amplifier controlled by pClamp11 software through an Axon Digidata 1550B system (Molecular Devices) (36). The bath solution contained 75 mM N-methyl-D-glucamine, 110 mM 2-(N-morpholino)-ethanesulphonic acid, 80 mM glucose, 1 mM MgCl_2_, 1 mM CaCl_2_, adjusted to pH_o_ 6.0 with methanesulfonic acid. The pipette solution contained 75 mM N-methyl-D-glucamine, 110 mM 2-(N-morpholino)-ethanesulphonic acid, 80 mM glucose, 1 mM MgCl_2_, 1 mM EGTA, adjusted to pH_i_ 6.0 with methanesulfonic acid. We performed recording in constant perfusion of the bath compartment to increase buffer turnover by convection, and the solution (pH_i_ = pH_o_ = 6.0) is used to minimize the effects of H^+^ accumulation on the local H^+^ concentration during the recording, and the *V*_*rev*_ of H_v_1 channels are closed to theoretical equilibrium Nernst potential (0 mV) for reversal potential (*V*_*rev*_) (Fig. S2 and Table S1). All measurements were performed at 22 ± 3 °C. Pipettes had 2 to 5 MΩ access resistance. Current traces were filtered at 1 kHz and analyzed with Clampfit11 (Molecular Devices) and Origin 2019 (OriginLab).

In the recording, proton currents were measured from a holding potential of −60 mV to a first prestep and a second test-step with rest intervals at −60 mV (37, 38). The proton channel conductance is determined by the equation: *G(V*_*test*_*) = (I*_*test*_*-I*_*tail*_*)/(V*_*test*_*-V*_*tail*_*)*, where *I*_*test*_ is the current at *V*_*test*_, measured at the end of the depolarization step, and *I*_*tail*_ is the current at *V*_*tail*_, measured at the beginning of the repolarization step. Currents after depolarization in the prestep were used to correct for current rundown. Steady-state activation *G-V* curves were fitted by the *Boltzmann* equation (39): *G/G*_*max*_ *= 1/(1 + exp(V*_*1/2*_*-V)/k)*, where *G/G*_*max*_ is the relative conductance normalized by the maximal conductance, *V*_*1/2*_ is the potential of half activation, *V* is the test pulse, and *k* is the slope factor. *k* is equal to *RT/ɀF*, where *ɀ* is the equivalent charge, *R* is the gas constant, *F* is *Faraday*’s constant, and *T* is temperature in *Kelvin*. Reported *V*_*1/2*_ and *k* values derived from the *Boltzmann* fits to data from multiple cells were used to assess the free energy difference at 0 mV (*ΔG*_*0*_) between the closed and open states of the activation, the *ΔG*_*0*_ was calculated according to the following: *ΔG*_*0*_ = *ɀFV*_*1/2*_. The channel opening time constant *Ʈ*_*act*_ and closing time constant *Ʈ*_*deact*_ values were calculated by fitting current traces with the single-exponential equation according to the following: *I(t) = I*_*0*_ *+ A(1-exp(-t/Ʈ))*, where *I(t)* represents the current at time point t, *I*_*0*_ is the initial current amplitude, *Ʈ* is the time constant.

## Data and statistical analysis

All data were presented as mean ± standard deviation. Significance between means was determined by Student’s *t* test. Electrophysiological parameters (*V*_*1/2*_*, Ʈ*_*deact*_*, Ʈ*_*deact*_, etc) were determined from each individual cell and used for comparison with two-tailed *t* test. *p* < 0.05 was considered to indicate a statistically significant difference and were indicated by ∗. *p* < 0.01 and *p* < 0.001 are signified by ∗∗ and ∗∗∗, respectively.

## Data availability

All data are contained within the article.

## Supporting information

This article contains supporting information.

## Conflict of interest

The authors declare that they have no conflicts of interest with the contents of this article.
