# Supplementary material for: Trp207 regulation of voltage-dependent activation of human Hv1 proton channel
Source: J Biol Chem. 2024 Jan 23;300(3):105674. doi: 10.1016/j.jbc.2024.105674 (PMC10875263; doi:10.1016/j.jbc.2024.105674)
Supplement: Supporting Figures [file mmc1.docx]

**SUPPLEMENTARY INFORMATION**

**Trp207 regulation of voltage dependent activation of human H_v_1 proton channel**

Lu Zhang, Xin Wu, Xinyu Cao, Khushi Rao, Liang Hong*

* Correspondence to: [hong2004@uic.edu](mailto:hong2004@uic.edu)


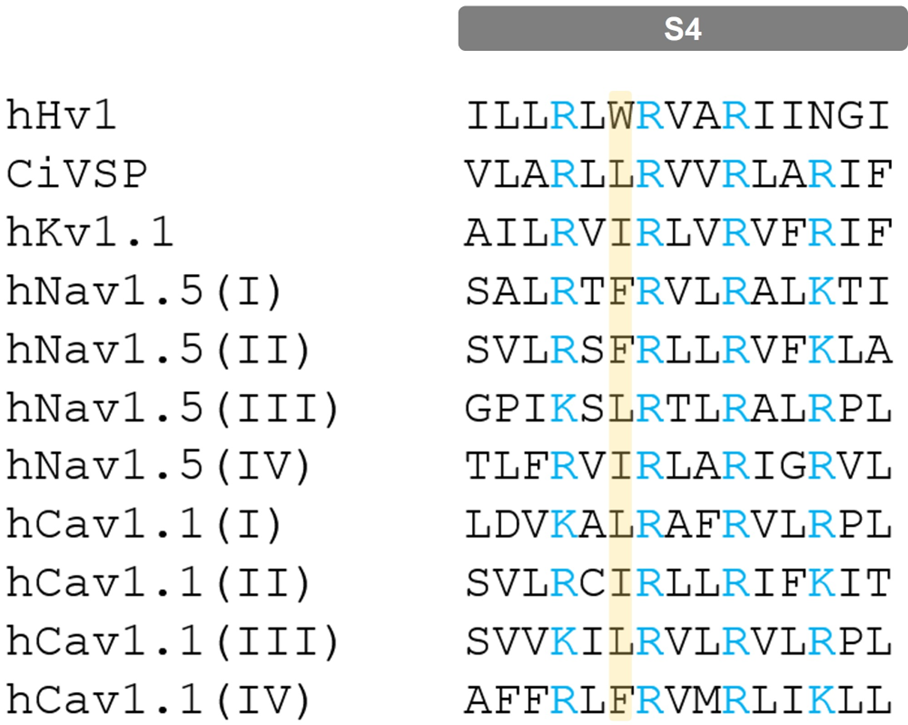


**Figure S1. The highly conserved tryptophan (W) of the H_v_1 family is not present in other voltage-sensing domains (VSDs).** Sequence alignment of the S4 transmembrane segment of VSDs from human H_v_1 proton channel (hH_v_1), ciona voltage sensitive phosphatase (CiVSP), human K_v_1.1 potassium channel (hK_v_1.1), human Na_v_1.5 sodium channel (hNa_v_1.5) domains I, II, III, IV, and human Ca_v_1.1 calcium channel (hCa_v_1.1) domains I, II, III, IV. The position of tryptophan in the S4 helix is highlighted in yellow font, positive charged residues are highlighted in blue.


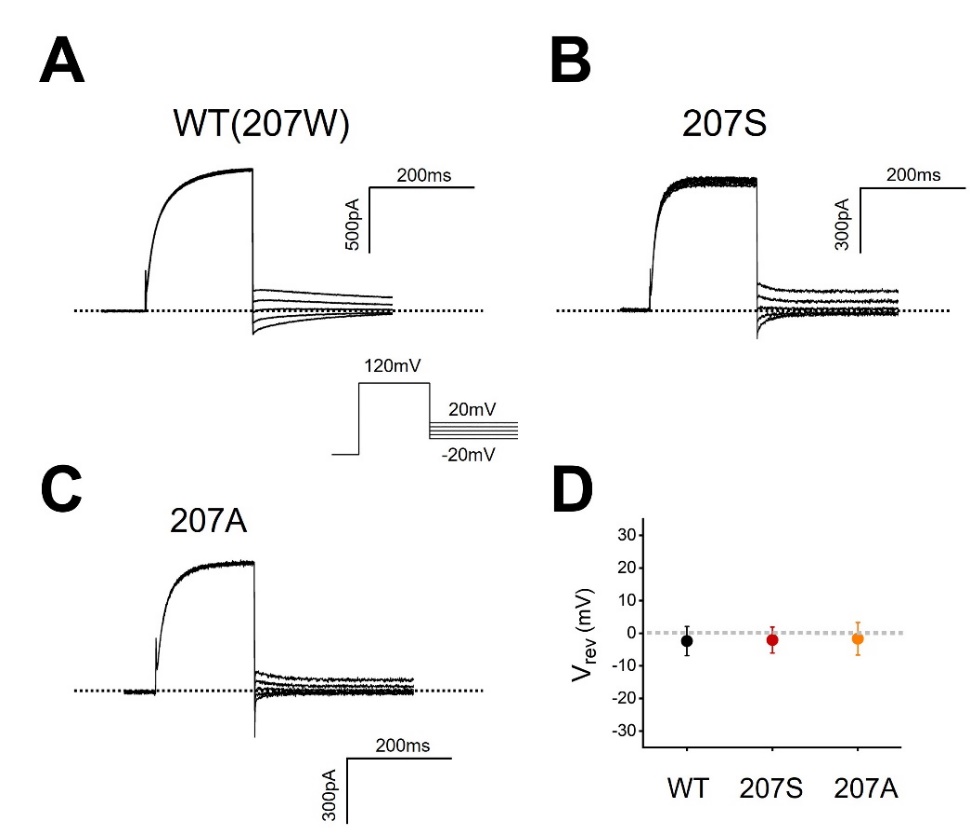


**Figure S2. Reversal potential measurements from monomer H_v_1 channels.** Representative currents in HEK293 cells expressing H_v_1 WT (**A**), 207S (**B**), and 207A (**C**). To record tail currents, depolarizing pre-pulse was given to +120 mV, after which the membrane potential was clamped from -20 mV to +20 mV. The black dash line represented 0 pA. pH_i_=pH_o_=6.0. (**D**) The theoretical equilibrium Nernst potential for reversal potential (*V_rev_*) is 0 shown by the gray dashed line. Error bars represent mean ± SD, n=4-6 for each group.


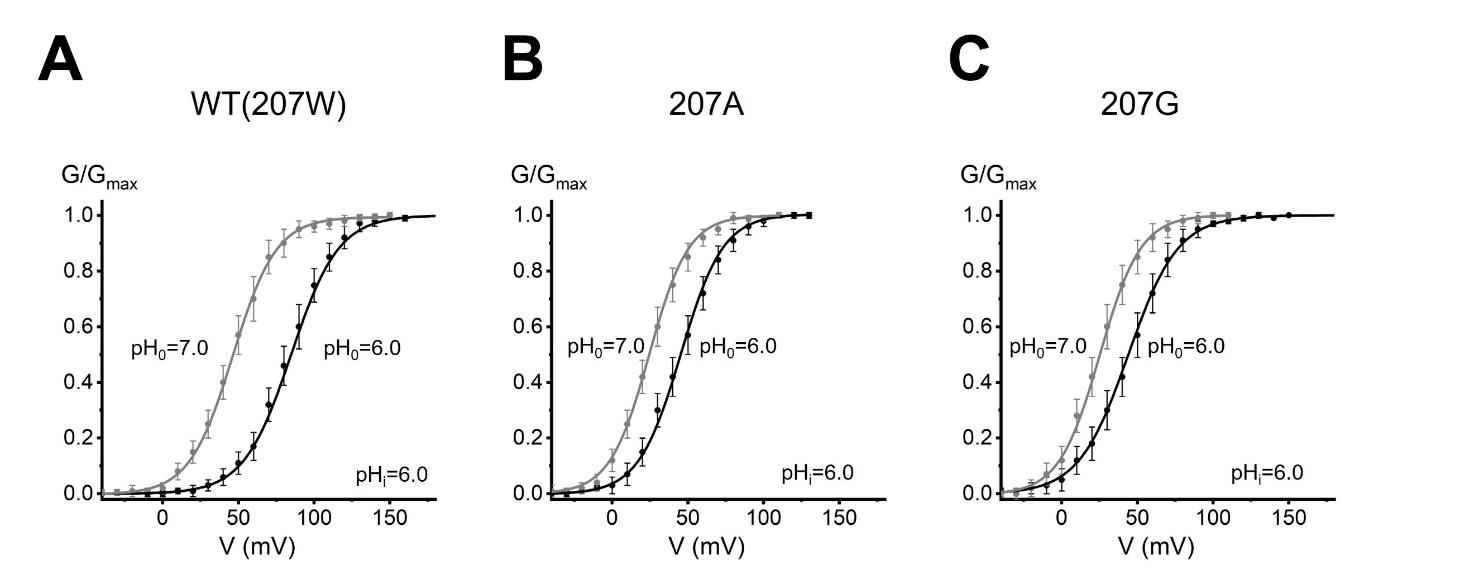


**Figure S3. Effects of W207 mutations on pH gradient gating.** A range of pH_o_=6.0-7.0 and pH_i_=6.0 were introduced to test *G-V* relationship for the Hv1 WT (**A**), 207A (**B**), 207G (**C**) in the HEK293 cells. The Data were fitted by *Boltzmann* function, n=4-8 for each group.
