# Supplementary material for: Trp207 regulation of voltage-dependent activation of human Hv1 proton channel
Source: J Biol Chem. 2024 Jan 23;300(3):105674. doi: 10.1016/j.jbc.2024.105674 (PMC10875263; doi:10.1016/j.jbc.2024.105674)
Supplement: Supporting Tables [file mmc2.docx]

**SUPPLEMENTARY INFORMATION**

**Trp207 regulation of voltage dependent activation of human H_v_1 proton channel**

Lu Zhang, Xin Wu, Xinyu Cao, Khushi Rao, Liang Hong*

* Correspondence to: [hong2004@uic.edu](mailto:hong2004@uic.edu)

**Table.S1 Effects of W207 mutations on the voltage-dependent activation of monomer H_v_1 channels.**

| ***Channel*** | ***n*** | ***V_1/2_(mV)*** | ***k(mV)*** | ***ΔG_0_(kjmol^-1^)*** | ***V_rev_(mV)*** |
| --- | --- | --- | --- | --- | --- |
| W207A | 6 | 47.8±2.9*** | 19.7±3.1* | 6.1±0.9*** | -1.7±4.8 |
| W207C | 4 | 51.1±7.7*** | 18.5±4.0 | 7.1±2.2** | -1.9±3.6 |
| W207D | 8 | 55.6±10.5*** | 16.8±2.7 | 8.5±2.6** | 2.8±4.1 |
| W207E | 6 | 65.0±11.5** | 18.3±2.4 | 8.9±1.7** | -5.1±6.3 |
| W207F | 6 | 74.3±9.6 | 18.8±2.3 | 9.9±1.8** | -1.5±4.7 |
| W207G | 5 | 45.0±5.5*** | 16.4±2.1 | 6.8±0.8*** | -2.4±3.5 |
| W207H | 4 | 69.8±12.4* | 14.3±2.5 | 12.7±4.9 | -2.9±2.8 |
| W207I | 4 | 60.8±5.7*** | 18.8±1.7 | 8.1±0.9** | 1.3±1.9 |
| W207K | 4 | 61.8±7.0** | 14.0±2.9 | 11.4±3.3 | -3.7±6.1 |
| W207L | 6 | 84.2±10.5 | 21.8±3.1** | 9.6±0.4** | 0.8±3.5 |
| W207M | 8 | 68.5±11.4** | 21.5±3.7** | 8.3±2.7** | -3.1±3.7 |
| W207N | 5 | 60.6±5.7*** | 19.2±3.2 | 8.0±1.6** | 2.1±2.6 |
| W207P | 5 | 72.6±9.2* | 20.4±2.3* | 9.0±2.0** | -1.4±3.2 |
| W207Q | 4 | 46.8±3.1*** | 19.5±2.6 | 6.0±0.9*** | 3.5±5.1 |
| W207R | 4 | 64.3±6.3** | 14.5±2.6 | 11.2±1.8 | -3.1±4.2 |
| W207S | 6 | 45.3±5.4*** | 19.7±2.7* | 5.8±1.3*** | -2.1±3.9 |
| W207T | 4 | 55.3±5.6*** | 18.5±1.3 | 7.4±0.6*** | 2.6±4.3 |
| W207V | 6 | 59.2±8.8*** | 18.2±3.2 | 8.2±1.4*** | -1.2±3.8 |
| 207W(WT) | 6 | 84.8±6.9 | 15.7±2.8 | 13.7±2.1 | -2.4±4.5 |
| W207Y | 4 | 80.3±3.9 | 16.3±2.5 | 12.4±1.9 | -3.9±5.2 |

*V_1/2_* and *k* values were derived from the *Boltzmann* fit and obtained from fitting conductance versus voltage (*G-V*) relations. *ΔG_0_* were determined by the equations shown in the methods. The reversal potential (*V_rev_*) was determined when channels were recorded at pH_i_=pH_o_=6.0. Error bars represent mean ± SD. Parameters (*V_1/2_, k, ΔG_0_, V_rev_*) between 207 mutation and WT (207W) were compared statistically using two-tailed test (* p < 0.05, ** p < 0.01, *** p < 0.001).

**Table S2. List of primers used for mutagenesis.**

| ***Constructs*** | ***Type*** | ***Primer Sequences (5' to 3')*** |
| --- | --- | --- |
| S224_stop_ | Forward | CTCAGTTAAGACACGTTCATAACGGCAACTCTTAAGG |
|  | Reverse | CCTTAAGAGTTGCCGTTATGAACGTGTCTTAACTGAG |
| W207A | Forward | CTGCTCCGGCTGGCTCGGGTGGCCCGG |
|  | Reverse | CCGGGCCACCCGAGCCAGCCGGAGCAG |
| W207C | Forward | CTCCGGCTGTGTCGGGTGGCCC |
|  | Reverse | GGGCCACCCGACACAGCCGGAG |
| W207D | Forward | CTGCTCCGGCTGGATCGGGTGGCCCGG |
|  | Reverse | CCGGGCCACCCGATCCAGCCGGAGCAG |
| W207E | Forward | GCTCCGGCTGGAGCGGGTGGCCCG |
|  | Reverse | CGGGCCACCCGCTCCAGCCGGAGC |
| W207F | Forward | GCTCCGGCTGTTCCGGGTGGCCCG |
|  | Reverse | CGGGCCACCCGGAACAGCCGGAGC |
| W207G | Forward | GCTCCGGCTGGGTCGGGTGGCCCG |
|  | Reverse | CGGGCCACCCGACCCAGCCGGAGC |
| W207H | Forward | CTGCTCCGGCTGCATCGGGTGGCCCGG |
|  | Reverse | CCGGGCCACCCGATGCAGCCGGAGCAG |
| W207I | Forward | CTGCTCCGGCTGATTCGGGTGGCCCGG |
|  | Reverse | CCGGGCCACCCGAATCAGCCGGAGCAG |
| W207K | Forward | GCTCCGGCTGAAGCGGGTGGCCCG |
|  | Reverse | CGGGCCACCCGCTTCAGCCGGAGC |
| W207L | Forward | CTCCGGCTGTTGCGGGTGGCCC |
|  | Reverse | GGGCCACCCGCAACAGCCGGAG |
| W207M | Forward | GCTCCGGCTGATGCGGGTGGCCCG |
|  | Reverse | CGGGCCACCCGCATCAGCCGGAGC |
| W207N | Forward | CTGCTCCGGCTGAATCGGGTGGCCCGG |
|  | Reverse | CCGGGCCACCCGATTCAGCCGGAGCAG |
| W207P | Forward | CTGCTCCGGCTGCCTCGGGTGGCCCGG |
|  | Reverse | CCGGGCCACCCGAGGCAGCCGGAGCAG |
| W207Q | Forward | GCTCCGGCTGCAGCGGGTGGCCCG |
|  | Reverse | CGGGCCACCCGCTGCAGCCGGAGC |
| W207R | Forward | CTCCGGCTGAGGCGGGTGGCCC |
|  | Reverse | GGGCCACCCGCCTCAGCCGGAG |
| W207S | Forward | GCTCCGGCTGAGTCGGGTGGCCCG |
|  | Reverse | CGGGCCACCCGACTCAGCCGGAGC |
| W207T | Forward | CTGCTCCGGCTGACTCGGGTGGCCCGG |
|  | Reverse | CCGGGCCACCCGAGTCAGCCGGAGCAG |
| W207V | Forward | CTGCTCCGGCTGGTTCGGGTGGCCCGG |
|  | Reverse | CCGGGCCACCCGAACCAGCCGGAGCAG |
| W207Y | Forward | GCTCCGGCTGTATCGGGTGGCCCG |
|  | Reverse | CGGGCCACCCGATACAGCCGGAGC |
